# Conquered from the Deep Sea? A New Deep-Sea Isopod Species from the Antarctic Shelf Shows Pattern of Recent Colonization

**DOI:** 10.1371/journal.pone.0049354

**Published:** 2012-11-07

**Authors:** Torben Riehl, Stefanie Kaiser

**Affiliations:** 1 Biocenter Grindel & Zoological Museum, University of Hamburg, Hamburg, Germany; 2 German Center for Marine Biodiversity Research, Senckenberg am Meer, Hamburg, Germany; University of Poitiers, France

## Abstract

The Amundsen Sea, Antarctica, is amongst the most rapidly changing environments of the world. Its benthic inhabitants are barely known and the BIOPEARL 2 project was one of the first to biologically explore this region. Collected during this expedition, *Macrostylis roaldi* sp. nov. is described as the first isopod discovered on the Amundsen-Sea shelf. Amongst many characteristic features, the most obvious characters unique for *M. roaldi* are the rather short pleotelson and short operculum as well as the trapezoid shape of the pleotelson in adult males. We used DNA barcodes (COI) and additional mitochondrial markers (12S, 16S) to reciprocally illuminate morphological results and nucleotide variability. In contrast to many other deep-sea isopods, this species is common and shows a wide distribution. Its range spreads from Pine Island Bay at inner shelf right to the shelf break and across 1,000 m bathymetrically. Its gene pool is homogenized across space and depth. This is indicative for a genetic bottleneck or a recent colonization history. Our results suggest further that migratory or dispersal capabilities of some species of brooding macrobenthos have been underestimated. This might be relevant for the species’ potential to cope with effects of climate change. To determine where this species could have survived the last glacial period, alternative refuge possibilities are discussed.

## Introduction

The Southern-Ocean benthos has been shaped by unique historical and environmental settings. The origin of the shelf fauna has been partly attributed to evolutionary polar emergence from the deep [1], [2] and to shelf connections with other continents that existed in times before the opening of the Drake Passage for deep-water currents about 33–34 mya [3]. Long-term isolation and *in situ* speciation have led to a highly endemic fauna on the shelf and slope surrounding Antarctica [4]. While homogenous abiotic conditions and circumpolar currents are likely explanations for the wide geographic and depth distributions of many taxa [5]–[7], there is evidence for geographic or bathymetric differentiation in others.

Recently, several closely-related lineages, previously overlooked due to morphological similarity (‘cryptic species’) have been discovered by means of molecular-genetic methods [8]–[15]. These suggest largely overestimated species’ distribution ranges but also underestimated diversity. The high diversity of the fauna has been attributed to Antarctica’s glaciological history [16]. A glacial diversity pump [17], [18] featuring repetitive expansions and subsequent retreats of glacial shields has possibly wiped out large proportions of the shelf fauna. It would have led to local extinctions, changes in population genetic structure [18] such as founder effects or bottlenecks and temporal isolation of remaining populations [19]. In addition, depth-related physiological barriers could play a role in their evolution as well [11], [20], [21]. The steep slopes as found in the bathyal region (i.e. between continental shelf break and continental rise) are characterized by strong abiotic and biotic gradients and habitat heterogeneity, thus facilitating population differentiation and ultimately speciation (i.e. depth-differentiation hypothesis) [22].

On the contrary, deep-water formation in some regions, upwelling in others and the absence of a thermocline might have facilitated polar emergence and submergence [5], i.e. the colonization processes from deep to shallow and vice versa [2], [23]–[25]. In support of this theory, typical elements of slope and abyssal communities can be encountered on the Antarctic continental shelf [26]–[30], such as deep-sea isopods. Abyssal and bathyal fauna might thus have emerged [1], [2], [31]–[33] and provided source populations for (re-) colonization of the shelf during interglacial periods [5], [34], although Barnes & Kuklinski [35] argue against this hypothesis, at least for bryozoans.

Isopods with a likely deep-sea origin have been frequently encountered around Antarctica [31]. One taxon for which the emergence scenario from the deep sea seems highly probable is the family Macrostylidae Hansen, 1916 [24], [36]–[38]. Macrostylids are a taxonomically well-defined and highly derived group. Currently, it is comprised of 82 described species with the majority of species recorded from abyssal depths in all oceans [39], many of which remain undescribed (Riehl, unpublished data). They have been described as a specialized endobenthic component of deep-sea macrofauna [40]–[42]. While the depth distribution of the family Macrostylidae has been found (uniquely) wide, between the shallow subtidal of 4 m (*Macrostylis spinifera* Sars, 1864 [43]) and hadal depths of almost 11,000 m (*M. mariana* Mezhov, 1993 [44]), almost no data are available to date on individual species’ spatial or depth distributions. However, the brooding mode of reproduction (direct development) and an infaunal or tubicolous lifestyle (i.e. digging or tube-dwelling) [41], [42], [45] are likely to lead to a very limited range of distribution. This is expected to promote genetic differentiation and allopatric fragmentation in populations, and finally speciation due to isolation by distance [46]–[48] (but see [49]–[52]). Prior to recent expeditions where macrostylids regularly occurred in samples from the Antarctic continental shelf [53] and a shallow seamount [54] they had rarely been reported from shallow depths [39].

The Amundsen Sea in the Southern Ocean is among the most rapidly changing regions on earth with unparalleled ice-sheet loss [55], due to warm-water advection [56]. Its fauna, though, has so far been barely studied. For the first time the benthic fauna of the Amundsen Sea was explored in detail in 2008 during the BIOPEARL 2 (Biodiversity, Phylogeny, Evolution and Adaptive Radiation of Life in Antarctica) cruise [53]. During this expedition, an isopod species of the family Macrostylidae was collected. It was identified as new to science and is described in this article. We furthermore assessed the genetic diversity in this species across sites differing in depth, spatial distribution and topography. According to the isolation-by-distance and depth-differentiation hypotheses, our assumption was that molecular data would reveal divergent lineages or potentially cryptic species. We hypothesized that the distribution of the haplotypes would be in congruence with topographic barriers and bathymetry. Finally, we intended to test our data for any indications for the presence of refuges and potential mechanisms where and how the species might have survived the Last Glacial Maximum [57]. A high level of nucleotide variability in sympatric specimens or across space and depth would indicate diversification, an old age of the population and in-situ survival. On the contrary, little variation would indicate a recent colonization from a refuge.

The possible existence of cryptic species within the samples could be ruled out. Instead, we found evidence for the presence of only one population with almost no nucleotide variability. Our data suggest that it is capable to maintain connectivity across space, depth and barriers. The observed pattern requires the assumption of a higher mobility than expected from Macrostylidae. The lack of nucleotide variability indicates further that the whole population is originating from a very small source population (bottle neck) and a recent colonization event can be hypothesized. Whether the species colonized the shelf from the slope, abyss or an ice-free refuge on the shelf could ultimately not be clarified.

## Results

### Systematics

**Asellota** Latreille, 1802 [58].

**Macrostylidae** Hansen, 1916 [36].

- Desmosomidae Sars, 1899 [59]
- Macrostylini Hansen, 1916, p. 74 [36]; Wolff, 1956, p. 99 [60]
- Macrostylinae Birstein, 1973 [61]
- Macrostylidae Gurjanova, 1933, p. 411; Menzies, 1962, p. 28, p. 127; Wolff, 1962; Birstein, 1970; Menzies and George, 1972, p. 79–81; Mezhov, 1988, p. 983–994; 1992, p. 69; Brandt 1992a, 2002, 2004; Kussakin, 1999, p. 336; Riehl and Brandt, 2010; Riehl *et al.,* 2012 [39], [62]–[73]

**Type genus.** *Macrostylis* Sars, 1864 [43].

- *Macrostylis* Sars, 1864 (Monotypic) [43]
- *Vana* Meinert, 1890 [74]
- *Desmostylis* Brandt, 1992 [69]

**Type species.** *Macrostylis spinifera* Sars, 1864 [43].

**Gender.** Female.

### Macrostylis Roaldi Riehl and Kaiser sp. nov

urn:lsid:zoobank.org:act:5ABAAC9D-3925-4A67-A009-84EA398C88AA.

#### Etymology

*Roaldi* is dedicated to the Norwegian explorer Roald Amundsen, eponym of the type locality, in order to mark the 100th anniversary of Amundsen as the first person to reach the geographic South Pole on December 14th 1911.

#### Type material examined

**Figure 1:**
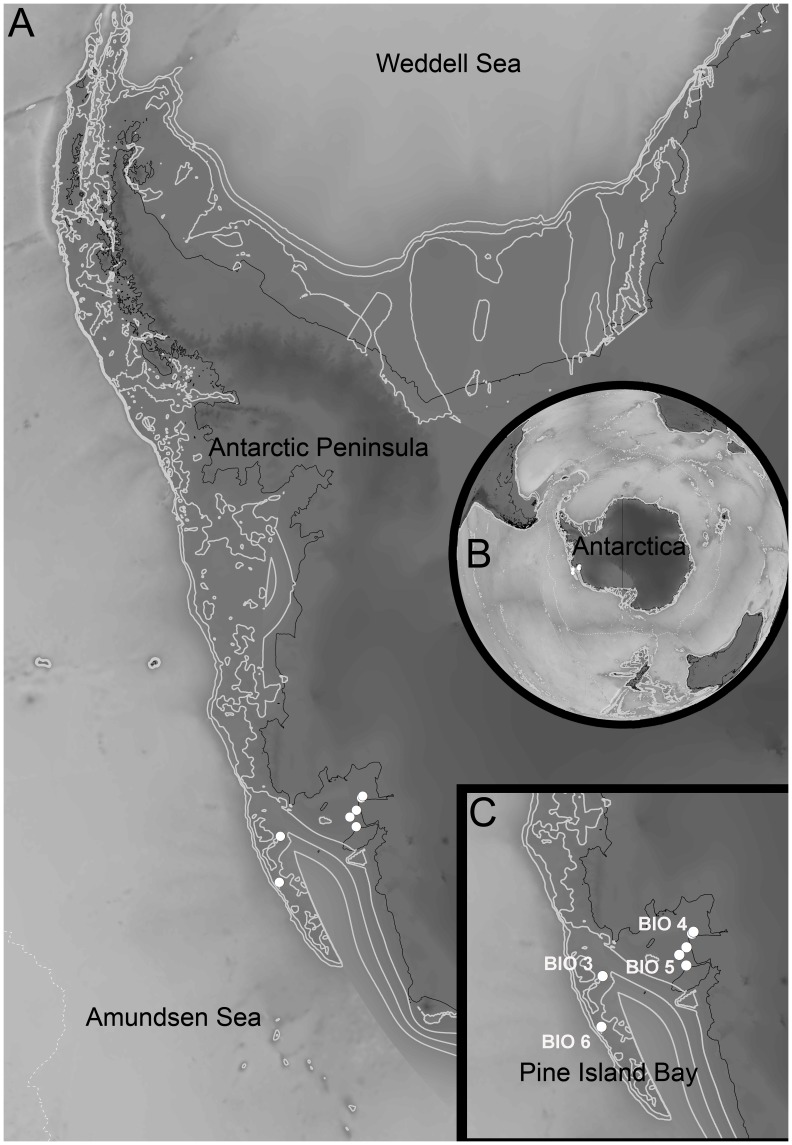
Type locality of *Macrostylis roaldi* sp. nov. A) Antarctic Peninsula with Amundsen Sea and Pine Island Bay, B) Antarctica, overview, C) Pine Island Bay, detail, with stations marked as white dots, grey dotted line marks the Polar Front, black contour lines indicate land mass boundaries, grey lines indicate 500 m depth contours.

See Table 1. **Type locality.** Pine Island Bay, Amundsen Sea, Southern Ocean (Fig. 1); for a complete list of records see Table 2. Abiotic data, such as sediment or bottom-water characteristics, are not available.

**Table 1:** Type material and further material examined for the description of *Macrostylis roaldi* sp. nov. with Process IDs of the “Barcode of Live Database” (BoLD) and GenBank accession numbers.

Stage	Sex	Collection no [ZMH-K]	Station no	BoLD Process ID	GenBank accession no			Condition
					COI	12S	16S	
**Holotype**								
Subadult, non-ovigerous	F	42994	BIO4-EBS-1A	TRII015-12	N/A	JX260302	JX260337	Partly dissected for DNA extraction, habitus and few appendages illustrated in situ
**Paratypes measured and illustrated in this study**								
Adult, non-ovigerous	F	42995	BIO04-EBS-1A	TRII014-12	N/A	JX260303	JX260338	Dissected for DNA extraction and illustration of appendages
Adult	M	42993	BIO04-EBS-1A	TRII016-12	JX260269	JX260301	JX260336	Dissected for DNA extraction and illustration of appendages
Juvenile	M	42997	BIO04-EBS-3B	TRII034-12	JX260258	JX260284	N/A	Partly dissected for DNA extraction and illustration of appendages
Adult, ovigerous	F	42998	BIO04-EBS-3B	TRII043-12	N/A	JX260275	JX260314	Partly dissected for DNA extraction and illustration of appendages
Adult	M	42999	BIO04-EBS-1A	TRII029-12	JX260259	N/A	JX260324	Partly dissected for DNA extraction, sputter-coated with carbon for SEM
Juvenile	M	43047	BIO04-EBS-1A	TRII017-12	JX260268	JX260300	JX260335	Partly dissected for DNA extraction
Adult, non-ovigerous	F	42999	BIO04-EBS-1A	TRII030-12	N/A	JX260288	JX260323	Partly dissected for DNA extraction, sputter-coated with carbon for SEM
**Type material used only for molecular analyses**								
Adult	M	43048	BIO04-EBS-3A	TRII010-12	JX260270	JX260304	JX260339	Partly dissected for DNA extraction
Adult	M	43049	BIO04-EBS-1B	TRII011-12TRII012-12	N/AN/A	N/AN/A	N/AN/A	Partly dissected for DNA extraction
Adult, ovigerous and mancae	F	42996	BIO04-EBS-1A	TRII018-12	N/AJX260267JX260266N/AJX260265JX260264JX260263JX260262JX260261	JX260299JX260298JX260297JX260296JX260295JX260294JX260293JX260292JX260291	JX260334JX260333JX260332JX260331JX260330JX260329JX260328JX260337JX260336	Partly dissected for DNA extraction; mancae used completely for DNA extraction, no voucher remains
Diverse	F	43050	BIO04-EBS-1A	TRII028-12TRII032-12TRII033-12	JX260260N/AN/A	JX260289JX260286JX260285	JX260325JX260322JX260321	Partly dissected for DNA extraction
Diverse	F+M	43051	BIO04-EBS-3B	TRII035-12TRII036-12TRII037-12TRII038-12TRII039-12TRII040-12TRII041-12TRII042-12	JX260257JX260256N/AN/AJX260255JX260254N/AN/A	JX260283JX260282JX260281JX260280JX260279JX260278JX260277JX260276	JX260320JX260319N/AJX260318N/AJX260317JX260316JX260315	Partlydissected for DNA extraction
**Further records**								
Non-ovigerous	F	42985	BIO05-EBS-2A	TRII001-12	JX260274	JX260313	JX260348	Partly dissected for DNA extraction
Adult	F+M	42986	BIO05-EBS-1A	TRII002-12TRII003-12TRII004-12	JX260273N/AN/A	JX260312JX260311JX260310	JX260347JX260346JX260345	Partly dissected for DNA extraction
Adult + juvenile	M	42987	BIO05-EBS-3B	TRII005-12TRII006-12	JX260272N/A	JX260309JX260308	JX260344JX260343	Partly dissected for DNA extraction
Adult + juvenile	F	42988	BIO03-EBS-1B	TRII007-12TRII008-12TRII009-12	N/AN/AJX260271	JX260307JX260306JX260305	JX260342JX260341JX260340	Partly dissected for DNA extraction
Adult, non-ovigerous	F	42989	BIO06-EBS-3A	TRII013-12	N/A	N/A	N/A	Partly dissected for DNA extraction

#### Type fixation

**Figure 2:**
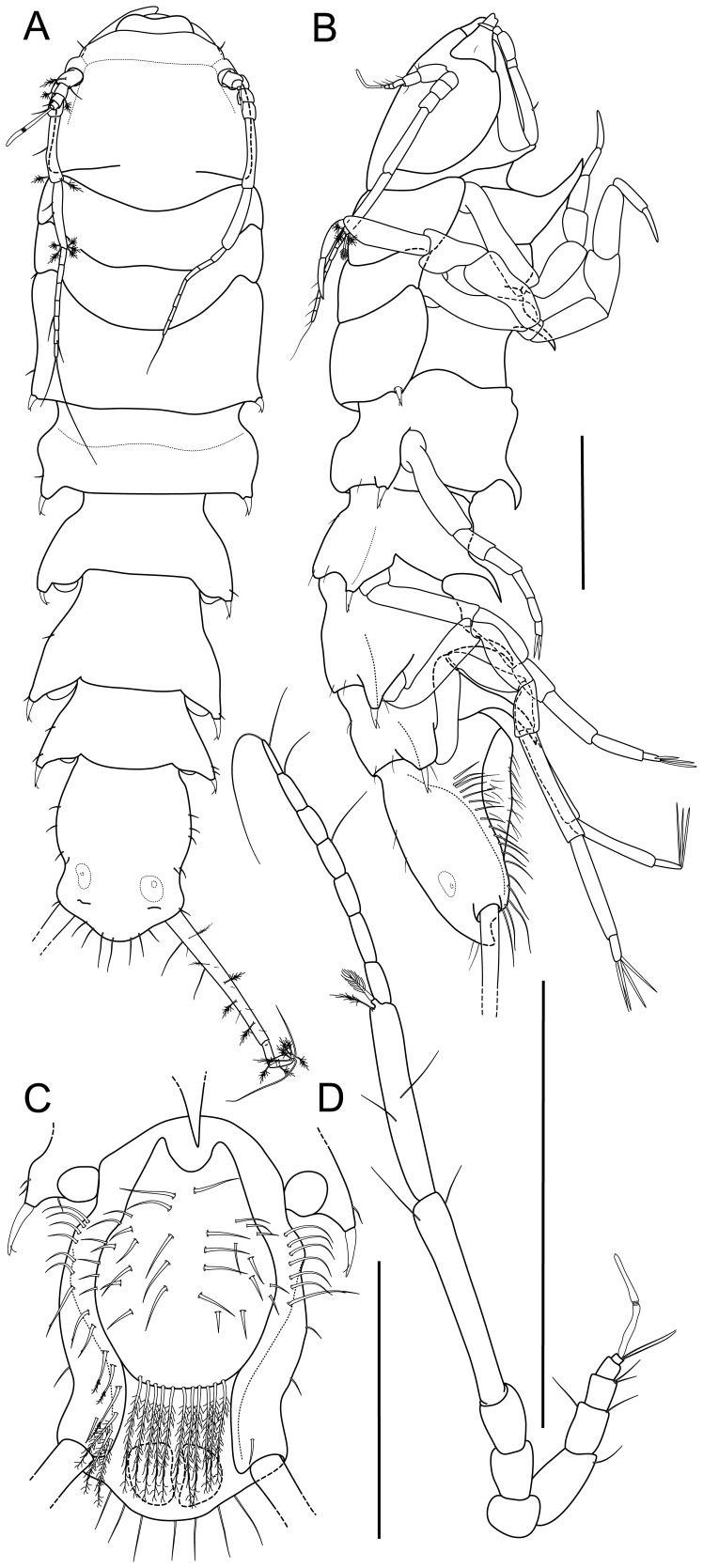
*Macrostylis roaldi* sp. nov., holotype female (ZMH-K42994). A) habitus, dorsal, B) habitus, lateral, C) pleotelson, ventral, D) antennula and antenna, lateral view, in situ. Scale bars = 0.5 mm.

Holotype: non-ovigerous female, 3.0 mm, ZMH-K 42994, designated here (Fig. 2).

#### Type material – Remarks

For DNA analyses, from all specimens 2–3 posterior pereopods were removed. See also Table 1.

#### Material examined for comparison

See Table 3.

### Description Female

**Figure 3:**
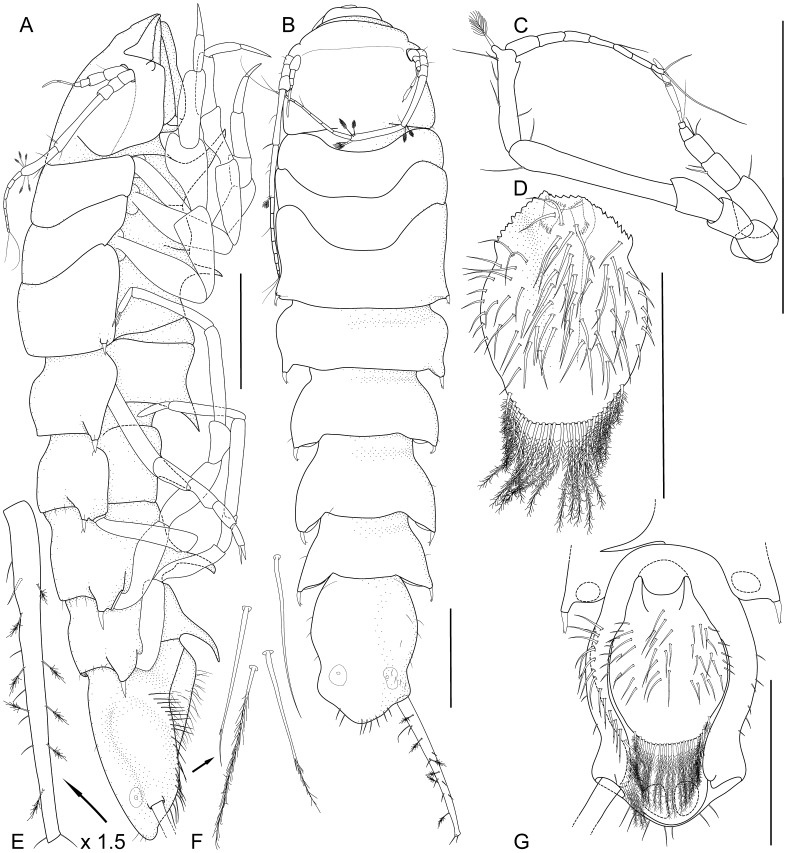
*Macrostylis roaldi* sp. nov., paratype female (ZMH-K42995). A) habitus, lateral, B) habitus, dorsal, C) antennula and antenna, lateral, in situ D) operculum, ventral, E) uropod protopod (endopod broken, missing), enlarged, F) setae from setal ridge, latero-ventrally on pleotelson in top-to-bottom order: simple, split, split and pappose, pappose, G) pleotelson, ventral. Scale bars = 0.5 mm.

**Figure 4:**
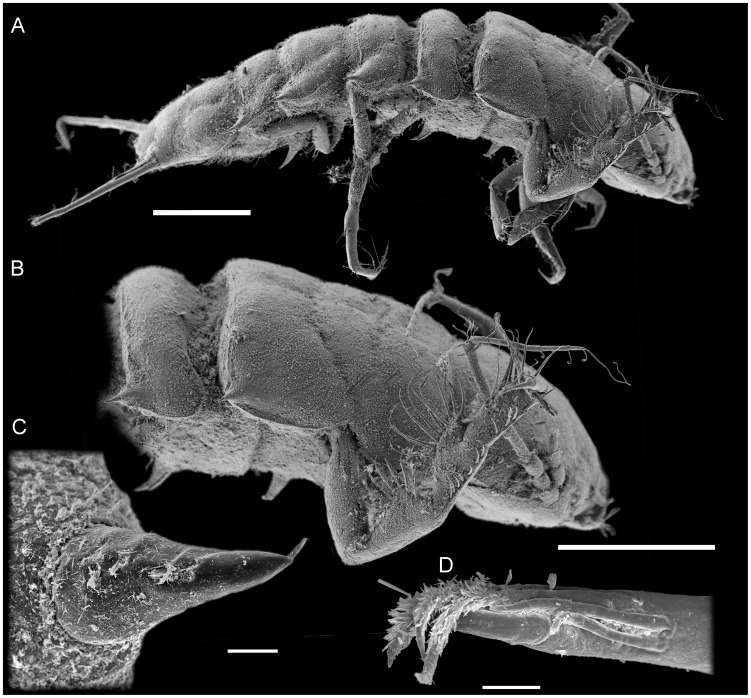
*Macrostylis roaldi* sp. nov., paratypes (ZMH-K42999), non-ovigerous female, SEM. A) habitus, dorsolateral, B) anteriot habitus, pereopod III, enlarged, C) robust, bifid, spine-like seta as on posterolateral corners of posterior tergites, D) pereopod III dactylus with claws and fringe-like sensillae, dorsolateral view when pereopod III in natural position. Scales: A, B = 0.5 mm, C, D = 0.01 mm.

Body ( Figs 2A–C , 3A–B, G , 4A–B 5A–B, D). Length 3.0–3.6 mm, 3.9–4.1 width, subcylindrical, tergite surfaces with scattered setae. Ventral spines. Pereonite 1 spine acute, prominent. Pereonite 3–6 spine acute, prominent, closer to posterior segment border. Pereonite 7 spine prominent. Imbricate ornamentation (IO). Cephalothorax-pleotelson IO weakly expressed, covering whole tergites, sternites and operculum. Cephalothorax. Length 0.88–0.90 width, 0.19–0.20 body length; frons in dorsal view concave, frontal ridge present, straight. Posterolateral setae present. Posterolateral margins blunt. Fossosome. Length 0.85–0.91 width, 0.22 body length. Lateral tergite margins in dorsal view forming almost uninterrupted line, ventral surface without keel; sternite articulations present, not fully expressed. Pereonite 1. Anterior margin concave; posterolateral setae simple. Pereonite 2. Posterolateral setae simple. Pereonite 3. Posterolateral margin produced posteriorly, tapering, culminating in articulation of posterolateral setae; setae bifid, robust, spine-like.

Pereonite 4. Width 1.1–1.2 pereonite 5 width, length 0.35–0.39 width; pereonal collum present. Lateral margins in dorsal view curved, concave in collum region, medially convex with greatest width, constricted anterior to posterolateral margin. Posterior tergite margin with 2 simple, not robust, flexibly articulating setae; setae short, not extending beyond posterolateral margin. Posterolateral margins produced posteriorly, tapering. Posterolateral setae bifid, robust, spine-like, articulating on pedestals (Fig. 4 A–C). Pereonite 5. Length 0.41–0.46 width. Posterior tergite margin with 4–6 simple, not robust, flexibly articulated setae; setae short, not extending beyond posterolateral margin. Posterolateral margins tapering. Tergite posterolateral setae bifid, robust, spine-like. Pereonite 6. Length 0.58–0.59 width. Posterior tergite margin with simple, not robust, flexibly articulating 4–8 setae; setae short, not extending beyond posterolateral angles. Posterolateral margin produced posteriorly, tapering. Tergite posterolateral setae bifid, robust, spine-like, articulating on pedestals. Pereonite 7. Length 0.45–0.46 width. Posterior tergite margin with 7–8 simple, not robust, flexibly articulating setae; setae short, not extending beyond posterolateral angles. Posterolateral margin produced posteriorly, tapering and subangular. Tergite posterolateral setae bifid, robust, spine-like, on pedestals.

**Figure 5:**
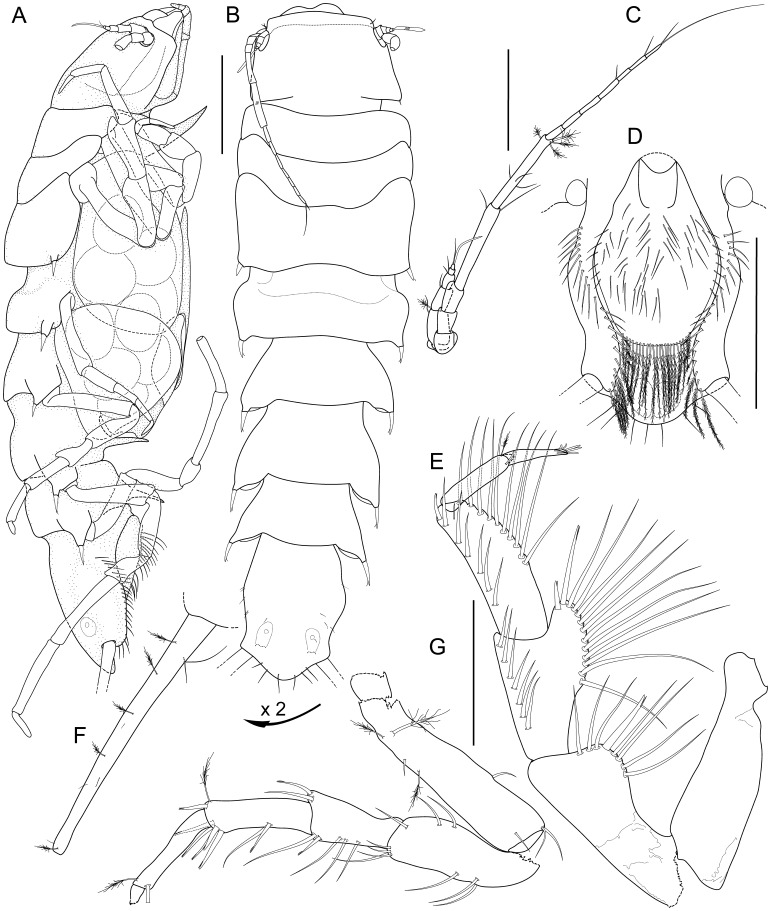
*Macrostylis roaldi* sp. nov., paratype ovigerous female (ZMH-K42998). A) habitus, lateral, B) habitus, dorsal, C) antennula and antenna, lateral, in situ D) pleotelson, ventral, E) pereopod III, F) uropod, enlarged, endopod broken, missing, G) pereopod V, basis, baso-ischial articulation and dactylus damaged. Scales A–B, D = 0.5 mm, C, E, G = 0.3 mm.

Pleotelson ( Figs 2C , 3G , 5D ). Constricted anteriorly to uropod articulations, ovoid, lateral margins convex, setal ridges visible in dorsal view, length 0.19–0.20 body length, 1.3–1.4 width, narrower than pereonite 7; statocysts present, dorsal slot-like apertures present, transverse across longitudinal axis, concave. Posterior apex convex, bluntly triangular. Posterior apex with 6–7 simple setae positioned on and around apex. Pleopodal cavity width 0.73 pleotelson width, preanal ridge width 0.43 pleotelson width. Anal opening terminal.

**Figure 6:**
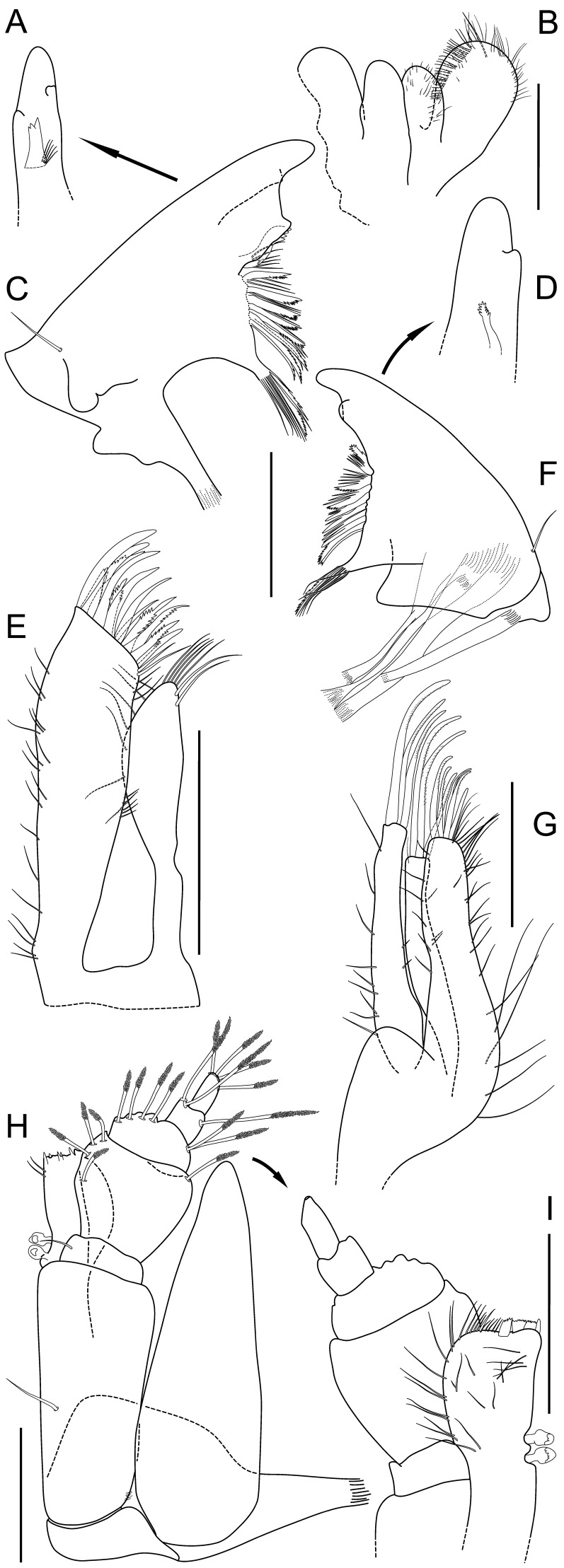
*Macrostylis roaldi* sp. nov., mouthparts: paratype adult male (ZMH-K42993, A-C, E-F, H-I), paratype female (ZMH-K42995, D, G). A) left mandible incisive process and lacinia mobilis, medial, B) paragnaths, C) left mandible, D) right mandible incisive process and lacinia mobilis, medial, E) maxillula, dorsal, F) right mandible, G) maxilla, dorsal, H) maxilliped, ventral, I) maxilliped endite and palp, dorsal, setae omitted. Scales = 0.1 mm.

Antennula ( Figs 2D , 3C , 5C ). Length 0.32 head width, 0.22 antenna length, width 1.0 antenna width. Articles decreasing in size from proximal to distal. Article 1 distinctly longer than wide, longest and widest, with 1 simple seta. Article 2 distinctly longer than wide, tubular, with 2 simple setae. Article 3 distinctly longer than wide, tubular, with 2 simple setae. Article 4 length subequal width, tubular. Article 5 squat, globular, with 2 simple setae. Terminal article with 1 aesthetasc, aesthetascs with intermediate belt of constrictions. Antenna ( Figs 2D , 3C , 5C ). Length 0.30 body length. Article 1 squat, globular. Article 2 squat, globular, longer than article 1. Article 3 elongate, longer than article 1. Article 4 longer than articles 1–3 together, distally with 2 simple setae. Article 5 shorter than article 4, distally with 2 broom setae. Flagellum with 7 articles. Mandibles ( Fig. 6A, C–D, F ). In medial view strongly narrowing from proximal to distal, sub-triangular, with lateral setae; left mandible incisor process distal margin flattened and curved (shovel-like), with 3 cusps, lacinia mobilis grinding or spine-like, adjacent to spine row without separating gap, with 3–4 cusps; right mandible incisior process bluntly rounded, with 2 cusps, lacinia mobilis grinding or spine-like, clearly smaller than left lacinia, adjacent to spine row without gap, with 10 cusps. Maxillula ( Fig. 6E ). Lateral lobe with 10 robust setae. Maxilla ( Fig. 6G ). Lateral lobe with 3 setae terminally, serrate; middle endite with 3 setae terminally, serrate; inner endite with 5 setae terminally, mostly serrate. Maxilliped ( Fig. 6H–I ). Basis length 3.3.3 width, medioventrally with seta present; epipod length 3.0 width, 1.1 basis length; palp wider than endite, article 2 wider than article 1, article 2 wider than article 3, article 1 shorter than article 3.

**Figure 7:**
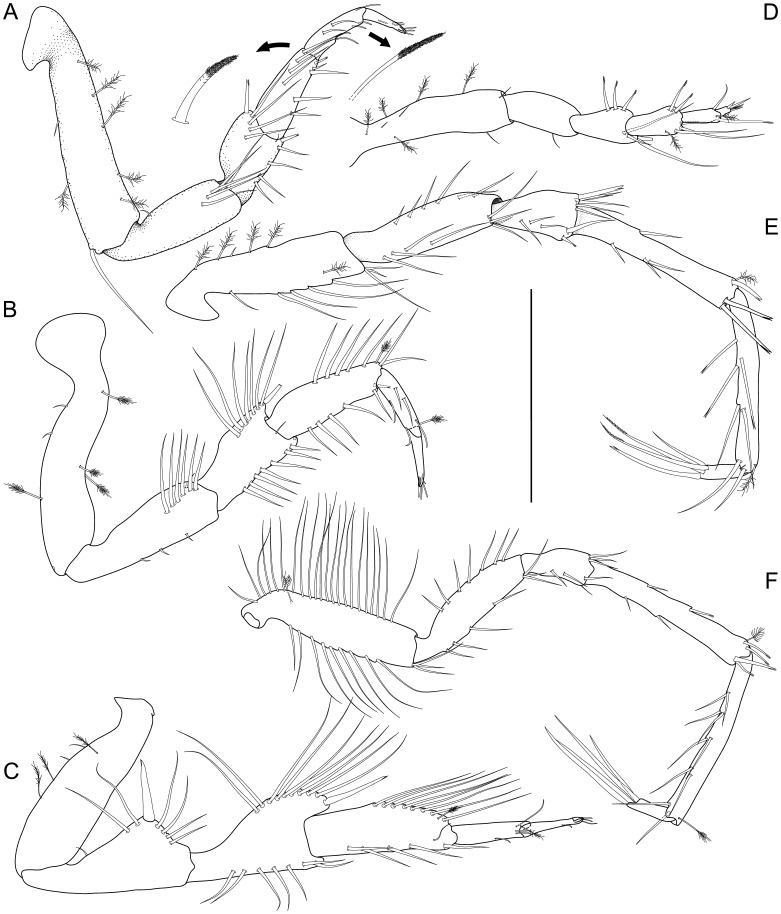
*Macrostylis roaldi* sp. nov., paratype female (ZMH-K42995), pereopods. A) Pereopod I, lateral, with enlarged setae (medially biserrate, distally fringe-like sensilla and distally fringe-like sensilla), B) pereopod II, lateral, C) pereopod III, lateral, D) pereopod IV, posterior, E) pereopod VI, medial, F) pereopod VII, medial. Pereopod V not shown, broken, missing. Scale = 0.5 mm.

Pereopod I ( Fig. 7A ). Length 0.42 body length. Ischium dorsal margin with 5–6 setae, simple, row of setae laterally to margin. Merus dorsal margin with 5 setae, 4 simple, 1 bifurcate, more robust, with dorsal row of setae laterally to margin; ventral margin with 5 medially biserrate, distally fringe-like sensillae. Carpus dorsally with 4 setae: 3 simple, 1 bifurcate, more robust. Dactylus distally with 3 sensillae. Pereopod II ( Fig. 7B ). Longer than pereopod I, length 0.46–0.47 body length. Ischium dorsally with 7 setae: 6 in row, simple, 1 distomedially, simple, with dorsal row of setae laterally to margin. Merus dorsally with 8 setae: 7 long, in row, simple, 1 short, more robust, split distally; ventrally with 8 distally fringe-like sensillae in row. Carpus dorsally with 8 setae: 5 medially biserrate, distally fringe-like sensillae in row, 1 broom, 2 simple distally; ventrally with 6 setae: 5 distally fringe-like sensillae in row, 1 split mediodistally. Dactylus distally with 3 sensillae. Pereopod III ( Figs 4B, D , 6E , 7C ). Length 0.47–0.48 body length. Ischium dorsal lobe triangular; proximally with 2–4 simple setae; apex with 1 prominent seta; apical seta robust, bifid, straight, spine-like; distally with 3–4 simple setae. Merus dorsally with 10–13 setae in row: 9–12 simple, 1 more robust, bifid distally; ventrally with 7 distally fringe-like sensillae in row. Carpus dorsally with 9–11 setae in row: 7–9 simple, 1 broom, 1 simple; ventrally with 6–8 setae: 5–7 distally fringe-like sensillae in row, 1 laterally, minute, simple. Dactylus with 3 sensillae.

Pereopod IV ( Fig. 7D ). Length 0.26 body length, carpus laterally flattened. Pereopod V ( Fig. 5G ). Ischium mid-dorsally with 2 simple setae; distodorsally with 1 short, simple seta, midventrally with 3 simple setae; distoventrally with 4 simple setae. Merus distodorsally with 2 setae: 1 simple, 1 split; midventrally with simple 3 setae; distoventrally with 2 setae: 1 short, split, 1 long, simple. Carpus distodorsally with 3 setae: 1 broom, 1 short, split, 1 long, simple; distoventrally with 5 split setae. Pereopod VI ( Fig. 7E ). Length 0.53 body length. Ischium dorsally with 6 simple setae in row; midventrally with 4 setae in row; distoventrally with 4 simple setae; middorsally with 6 simple setae in row. Merus middorsally with setae absent; distodorsally with 6 setae: 2 simple, 1 prominent, split and more robust, 4 simple; midventrally with 3 simple setae in row; distoventrally with 2 setae: 1 simple, 1 spine-like, split. Carpus middorsally with 1 seta; distodorsally with 2 setae: 1 broom, 1 bifurcate; midventrally with 3 setae; distoventrally with 2 split setae. Pereopod VII ( Fig. 7F ). Length subequal to pereopod VI length, 0.52 body length; basis length 3.2–4.2 width, dorsal margin row of elongate setae present, setae longer basis width, 22 altogether, ventral margin row of elongate setae present, setae longer basis width, 9–10 altogether. Ischium length 3.7 width, middorsally with 7 setae; midventrally with 4 setae in row; distoventrally with 3 setae. Merus length 2.4 width, distodorsally with 3 setae, midventrally with 2 setae, distoventrally with 2 setae. Carpus length 6.0 width, mid-dorsally with 2 bifid or split setae; distodorsally with 3 setae: 2 bifid or split, 1 broom; mid-ventrally with 2 setae; distoventrally with 2 setae: 1 short, bifid or split, 1 long, bifid or split. Propodus length 8.6 width. Dactylus length 3.3 width.

Operculum ( Fig. 3D ). Stout, length 1.2 width, 0.7 pleotelson dorsal length; apical width 0.69 operculum maximal width; distally not reaching anus, ovoid, ventrally keeled. With lateral fringe consisting of 6–7 setae, lateral fringe of setae distinctly separate from apical row of setae. With 22 pappose setae on apex, completely covering anal opening.

**Figure 8:**
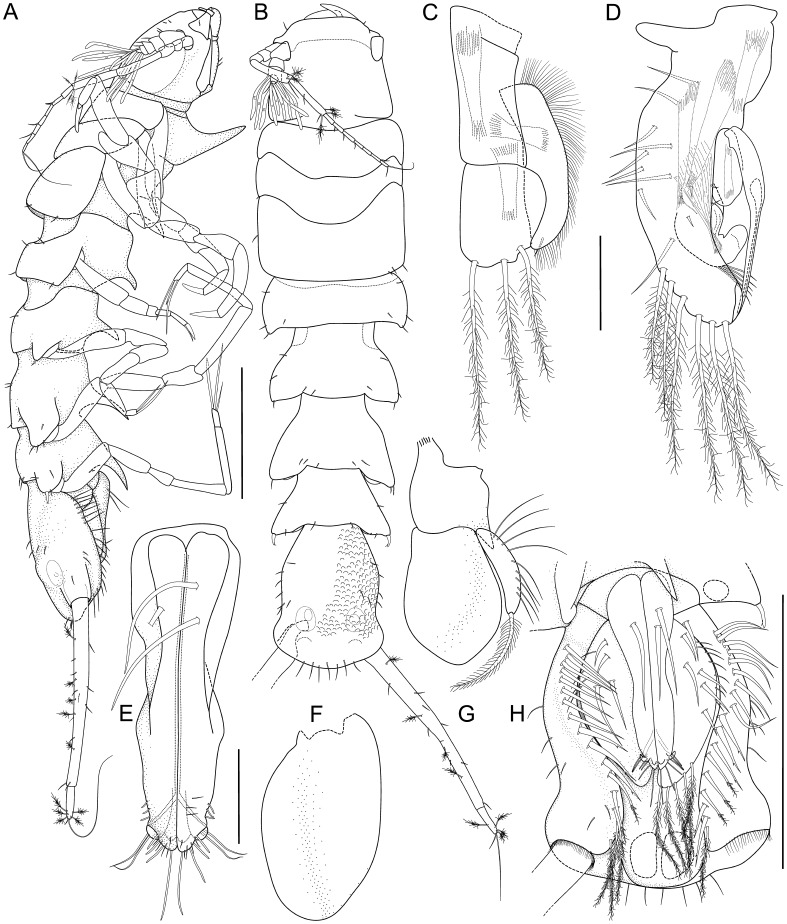
*Macrostylis roaldi* sp. nov., paratype adult male (ZMH-K42993), habitus and pleopods. A) habitus, lateral, B) Habitus, dorsal, C) pleopod III, dorsal, D) pleopod II, dorsal, E) pleopod I, ventral, F) pleopod V, ventral, G) pleopod IV, ventral, H) pleotelson, ventral. Scales: A, B, H = 0.5 mm; C-G = 0.1 mm.

Pleopod III ( Fig. 8C ). Length 2.4 width, protopod length 2.3 width, 1.6 pleopod III length; exopod with fringe of fine setae, shorter than pleopod III exopod width, with 1 simple seta subterminally, exopod length 0.63 pleopod III length. Pleopod V ( Fig. 8F ). Present. Uropod ( Figs 2A , 3B, E , 5F ). Inserting on pleotelson on posterior margin; length 1.2 pleotelson length; protopod length 8.7–10.4 width, 0.93–1.0 pleotelson length, protopod distal margin blunt, endopod insertion terminal; endopod length 3.5 width, 0.27 protopod length, endopod width at articulation subsimilar protopod width.

### Description Adult Male

**Figure 9:**
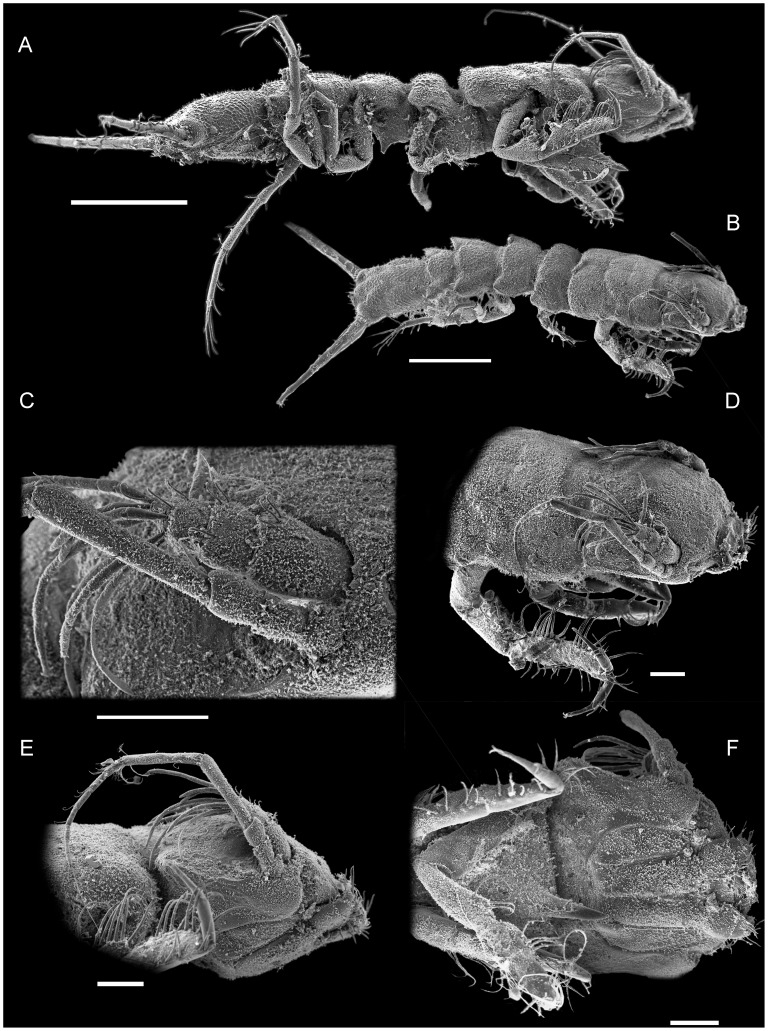
*Macrostylis roaldi* sp. nov., paratypes (ZMH-K42999), adult male, SEM. A) habitus, lateral, B) habitus, dorsolateral, C) antennula, antenna, basal segments, D) cephalothorax, dorsolateral, E) cephalothorax, antenna, lateral, F) cephalothorax, mouthparts, ventral. Scales: A, B = 0.5 mm, C–F = 0.1 mm.

Body ( Figs 8A–B, H , 9A–B ). More elongate than female, subcylindrical, elongate, length 2.4 mm, 4.4 width. Imbricate ornamentation (IO). Cephalothorax IO weakly expressed, covering whole tergite and sternite, pereonite 3–pleotelson IO strongly expressed, covering whole tergite, sternite and pleopods II. Cephalothorax. Frontal ridge present, straight between insertions of antennulae; length/width ratio subequal to female, length 0.92 width, 0.17 body length; posterolateral corners rounded. Fossosome. Length/width ratio greater than in female, length 1.0 width, length/body-length ratio subequal to female, not keeled. Pereonite 2. Posterolateral setae present, simple, not robust, without pedestals. Pereonite 3. Posterolateral setae present, simple, not robust, flexibly articulated. Length in male 0.29 width.

Pereonite 4. Pereonal collum present, medially straight. Lateral margins in dorsal view convex; posterolateral margins produced posteriorly. Posterolateral setae present, not robust, simple, flexibly articulated. Pereonite 5. Posterior tergite margin as in female. Produced posteriorly, rounded. Simple, not robust, flexibly articulated. Pereonite 6. Produced posteriorly, rounded. Simple, not robust.

Pleonite 1 ( Fig. 8H ). Sternal articulation with pleotelson present. Pleotelson. In dorsal view constricted anterior to uropod articulation trapezoid, widening posteriorly, lateral margins straight, length/width ratio in male subequal to female, 0.22 body length, width less than pereonite 7 width, tergite dorsal surface in posterior view with axial ridge and 2 lateral fields. Posterior apex convex, very flat, almost straight, pleopodal cavity width 0.62 pleotelson width, preanal ridge width 0.33 pleotelson width.

Antennula ( Figs 8A, B , 9C–E ). Length 0.26 head width, 0.25 antenna length, width 1.75 antenna width; terminal article with 2–3 aesthetascs, penultimate article with 7–8 aesthetascs (Fig. 9C), aesthetascs with intermediate belt of constrictions. Article 1 elongate, longest and widest, with 3 simple setae, 1 broom seta. Article 2 squat, globular, shorter than article 1, with 4 simple setae, 1 broom seta. Article 3 squat, globular, shorter than article 1, with 2 simple setae. Article 4 squat, globular, shorter than article 1, with 1 simple seta. Article 5 squat, globular, shorter than article 1, with 1 simple seta. Antenna ( Figs 8A–B , 9C–E ). Length 0.33 body length. Flagellum of 7 articles. Article 1 squat, globular. Article 2 squat, globular, shorter than article 1. Article 3 elongate, longer than article 1. Article 4 longer than articles 1–3 together, distally with 1 simple seta, 2 broom setae. Article 5 shorter than article 4. 4 broom setae.

**Figure 10:**
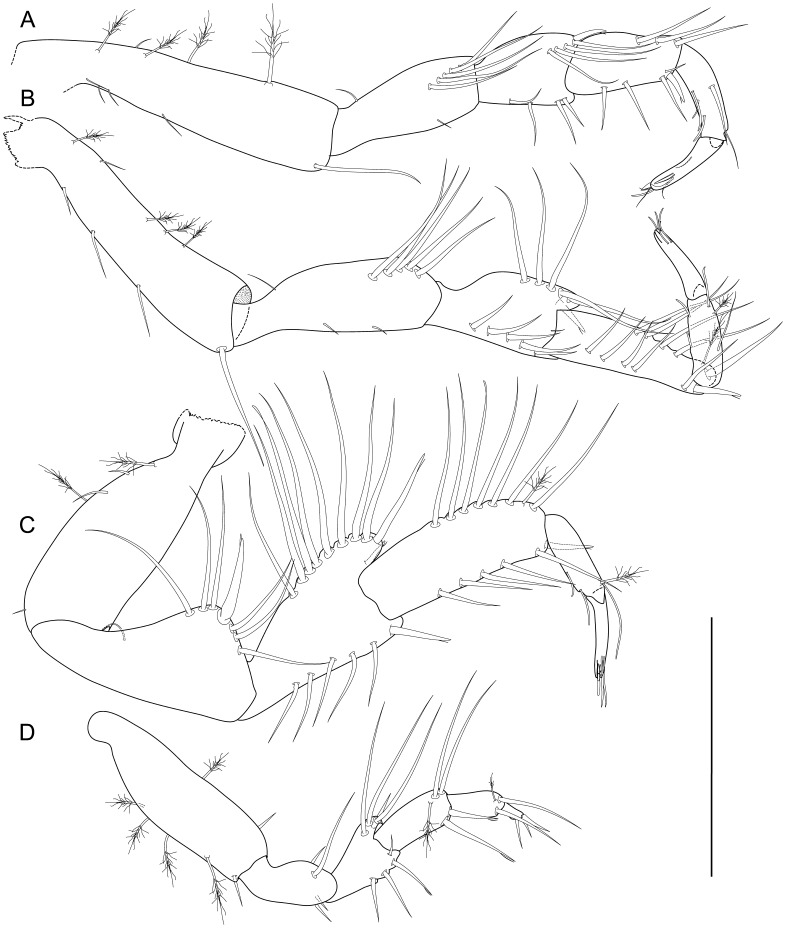
*Macrostylis roaldi* sp. nov., paratype adult male (ZMH-K42993), anterior pereopods. A) pereopod I, lateral, B) pereopod II, lateral, carpo-propodal articulation twisted, C) pereopod III, lateral, D) pereopod IV, posterior. Scale = 0.3 mm.

Pereopod I ( Fig 10A ). Length 0.39 body length. Merus setation as in female. Carpus dorsally with 3 simple setae in row; ventrally with 5 setae: 3 simple, in row, 1 small, simple, distolaterally, 1 spine-like, robust, split distoventrally. Pereopod II ( Fig. 10B ). Length/body-length ratio sexually dimorphic; length 0.44 body length. Ischium dorsally with 5 setae, simple, long, with dorsal row of setae shifted laterally. Merus dorsally with 6 setae: 5 simple, long in row, 1 spine-like, robust, bifid distomedially; ventrally with 5 simple setae. Carpus dorsally with 6 setae: 5 simple, long in row, 1 broom subdistally; ventrally with 6 setae: 5 simple in row with larger distance between setae 4 and 5, 1 spine-like, robust, bifid distomedially. Pereopod III ( Fig 10C ). Ischium sexually dimorphic; triangular, proximally with 3 simple setae. Ischium apex with 1 prominent seta; apical seta robust, spine-like, straight, bifid. Distally with 3 simple setae. Merus dorsally with 10 setae: 8 long, simple in row, 1 slightly more robust, split distally, 1 short, spine-like, robust bifid seta distomedially; ventrally with 6 setae: 5 simple in row, 1 slightly more robust, split distally. Carpus dorsally with 8 setae: 7 long, simple in row, 1 broom subterminally; ventrally with 6 setae: 5 simple in row, 1 slightly more robust, split distally.

**Figure 11:**
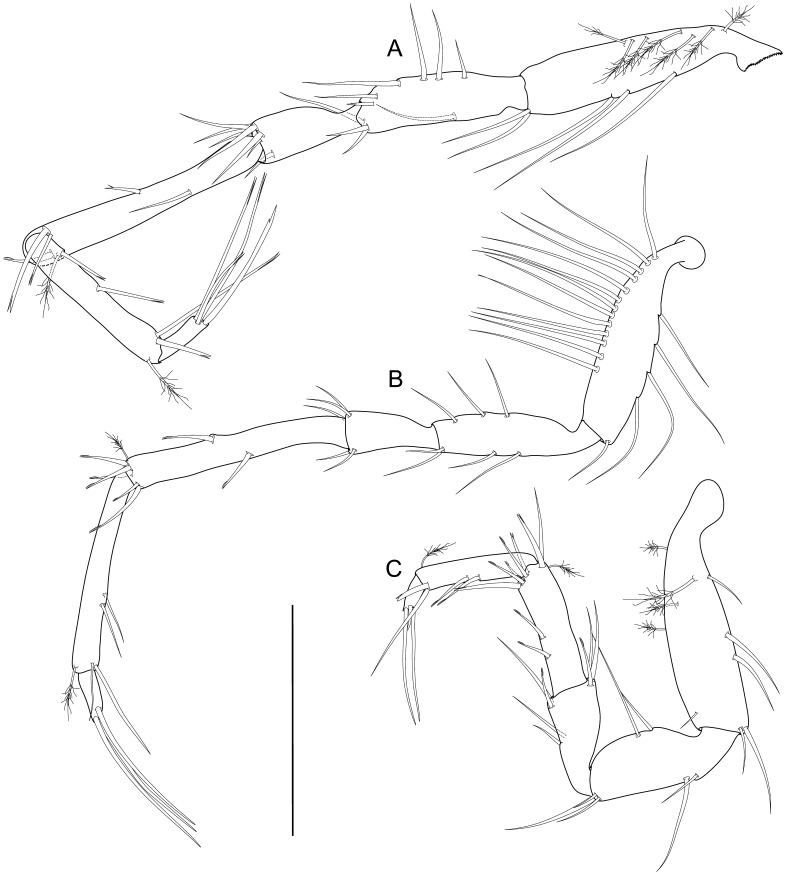
*Macrostylis roaldi* sp. nov., paratype adult male (ZMH-K42993), posterior pereopods. A) pereopod VI. lateral, B) pereopod VII, lateral, C) pereopod V, lateral. Scale = 0.3 mm.

Pereopod IV ( Fig. 10D ). Length 0.24 body length. Pereopod V ( Fig. 11C ). 0.39 body length. Ischium middorsally with 2 long, simple setae. Ischium distodosally with setae absent. Ischium midventrally with 2 setae, 1 short, simple, 1 long, simple, distoventrally with 3 setae: 2 short, simple, 1 long, simple. Merus distodorsally with 3 setae: 1 split, 2 simple, long; midventrally with 2 simple setae; distoventrally with 2 setae: 1 short, split, 1 long, simple. Carpus setation as in female. Pereopod VI ( Fig. 11A ). Ischium dorsally with 6 setae: 5 simple, in row, 1 short, split; midventrally with 1 simple seta; distoventrally with 3 setae: 2 short, simple, 1 long, simple. Merus distodorsally with 6 simple setae. Merus midventrally with setae absent. Distoventrally with 1 simple seta. Carpus middorsally with 1 split seta, distodorsally with 2 setae: 1 short, split, 1 long, split; midventrally with 1 simple seta, distoventrally with 2 setae: 1 broom, 1 split. Pereopod VII ( Fig. 11B ). Length 0.49 body length, length less than pereopod VI length, segment L/W ratios sexually dimorphic; basis length 3.9 width, dorsal margin row of elongate setae sexually dimorphic, setae longer basis width, 13 altogether, ventral margin row of elongate setae sexually dimorphic, setae longer basis width, 4 altogether; ischium length 3.3 width, middorsally with 3 simple, long setae; midventrally with 2 simple, long setae; distoventrally with 2 simple setae. Merus length 2.0 width; distodorsally with 3 simple setae, distoventrally with 2 simple setae; carpus length 7.3 width. Carpus mid-dorsally with 1 split seta; distodorsally with 4 setae: 1 broom, 3 split; midventrally with 1 split seta, distoventrally with 2 setae: 1 short, split, 1 long, split. Propodus length 6.5 width. Dactylus length 3.5 width.

Pleopod I ( Fig 8E, H ). Length 0.63 pleotelson length, lateral horns not extending distally beyond medial lobes, distally with 9 sensillae, ventrally with setae present, 1–2 setae proximally, longer than pleopod I width, 8 minute setae distally. Pleopod II ( Fig. 8D ). Protopod apex rounded, with 7 setae on proximal lateral margin; with 5 pappose setae distally. Endopod distance of insertion from protopod distal margin 0.59 protopod length. Stylet weakly curved, not extending to distal margin of protopod, length 57.9 protopod length. Uropod ( Fig. 8A–B ). Length 1.5 pleotelson length; protopod length/width ratio subequal to female, 8.9 width, with endopod inserting terminally; endopod/protopod length ratio less than in female, endopod length 0.15 protopod length, endopod length 3.7 width, width subequal protopod width.

**Remarks.** The specimens included in this study were retrieved from eight stations with a minimum distance between stations of about 0.6 km and a maximum distance of roughly 300 km (Fig. 1, Table 1). The depth range lies between 478 and 1,486 m and thus the Pine Island Bay area features potentially significant physical barriers to dispersal (see maps provided by Lowe & Anderson [75] and Kaiser et al. [53]). The collection at hand comprises 47 specimens, 1 manca, 31 females and 15 males.

The manca is 1.5 mm in length: sex indeterminable; pereonite 7 very small, posterolateral protrusions and setae both absent; antennula with 1 aesthetasc; pereopod III ischium dorsal lobe proximally with setae absent, distally 1 seta present. Pereopod VII absent.

Four male stages were identified and could be differentiated mainly based on the stage of development of the pereopod VII and pleopod I:

Two specimens (1.6 and 1.8 mm length) were identified as first male stage: pereonite 7 small with posterolateral protrusions and setae both absent; antennula eutrophied, with 1 aesthetasc; pereopod III ischium dorsal lobe proximally with 1 seta, and distally with 1 seta; pereopod VII developing, shorter than pereopod VI, without setae; strongly flexed at basis-merus articulation; both pereopods VII adjoined between merus and dactylus and extending along midline of body to the distal tip of pleopod I; pleopod I posteriorly projecting about 60% of pleopod II length.

Three specimens (2.0–2.1 mm length) have been found belonging to a second male stage: pereonite 7 small, posterolateral protrusions and setae both present, disproportionally large; antennula eutrophied, with 1 aesthetasc; pereopod III ischium dorsal lobe proximally with 1–2, and distally with 2–3 setae; pereopod VII shorter (about 60%) than pereopod VI, with setae present and in normal position and orientation; pleopod I projecting posteriorly to about 80% of pleopod II length.

Four specimens could be allocated to a third male stage (1.9–2.7 mm length): pereonite 7 fully developed, little shorter than pereonite 6, with posterolateral protrusions and setae both subequal to pereonite 6; antennula eutrophied, with 1 aesthetasc; pereopod III ischium dorsal lobe proximally with 1–3, distally with 2–3 setae; pereopod VII fully developed, little shorter and more slender than pereopod VI; pleopod I projecting posteriorly to about 90% of pleopod II length (as in adult) (Fig. 12).

**Figure:**
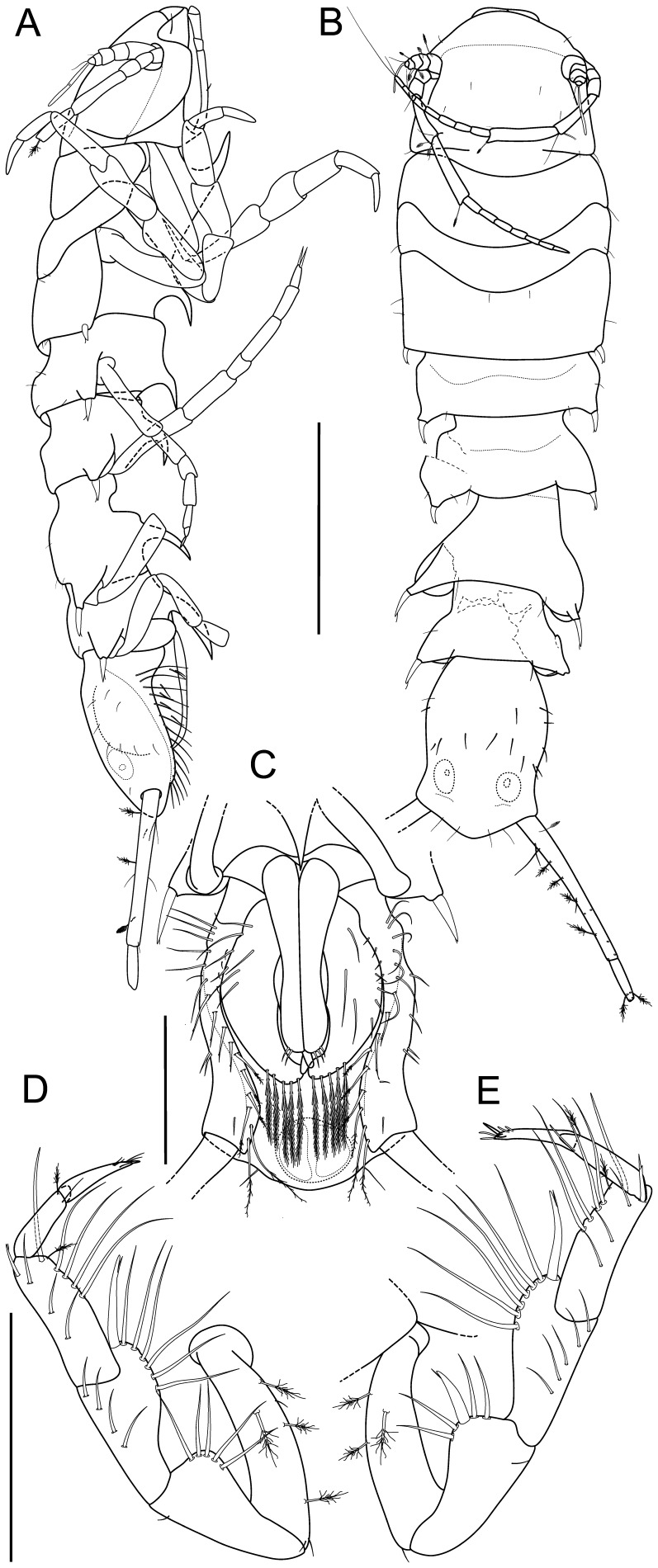
*Macrostylis roaldi* sp. nov., paratype juvenile male (ZMH-K42997). A) habitus, lateral, B) habitus, dorsal, posterior pereonites damaged, C) pleotelson, ventral, D) left pereopod III, E) right pereopod III. Scales: A, B = 0.5 mm; C = 0.2 mm; D, E = 0.3 mm.

Six male were found in adult stage (2.1–2.5 mm length): pereonite 7 fully developed, little shorter than pereonite 6, with posterolateral protrusions and setae both subequal to pereonite 6; antennula eutrophied, with 6–9 aesthetascs; pereopod III ischium dorsal lobe proximally with 2–3, distally with 2–4 setae; pereopod VII fully developed, little shorter and more slender than pereopod VI; pleopod I distally differentiated, projecting posteriorly to about 90% of pleopod II length.

Three females belong to the smallest female stage identified (2.2–2.5 mm): pereonite 7 small, posterolateral protrusions and setae both present and disproportionally large; antennula with 1 aesthetasc; pereopod III ischium dorsal lobe proximally with 1–2, and distally with 1–2 setae; pereopod VII shorter (about 60%) than pereopod VI, with setae present and in normal position and orientation.

21 females (2.2–3.7 mm length) could not clearly be allocated to a stage as developmental stages of single characters tend to overlap strongly and categories mix: pereonite 7 almost fully or fully developed, little or clearly shorter than pereonite 6, with posterolateral protrusions and setae both subequal to pereonite 6; antennula with 1 aesthetasc; pereopod III ischium dorsal lobe proximally with 2–4, distally with 2–4 setae; pereopod VII of 60% peropod VI length or fully developed, little shorter and more slender than pereopod VI, with setae present.

Four ovigerous females were found (3.2–3.8 mm length): pereonite 7 fully developed, little shorter than pereonite 6, with posterolateral protrusions and setae both subequal to pereonite 6; antennula not eutrophied, with 1 aesthetasc; pereopod III ischium dorsal lobe proximally with 3–4, distally with 3–4 setae; pereopod VII fully developed, little shorter and more slender than pereopod VI.

Female stages I and II were not found. Setal counts on the pereopod III ischium dorsal lobe often varied between left and right side of the same individual. The proximal setal row had one seta less on the right side in six specimens, and one seta more in four specimens. The distal row featured one seta more on the right side in seven cases and one less in four cases.

### Development

Setal counts on the pereopod III dorsal lobe are not normally distributed. Therefore, a non-parametric spearman correlation was conducted. We found a significant correlation between body length (mm) and total number of setae of the right and left pereopods (spearman correlation right: rS = 0.82, p<0.0001, n = 46; left: rS = 0.83, p<0.0001, n = 37).

### Molecular Results

Sequence fragments of the mitochondrial COI gene were obtained from 22 macrostylid specimens resulting in a 657 bp alignment with two single variable sites occurring in a single specimen (two haplotypes are separated by two point mutations: transition (guanine ← → adenine) at position 244, transversion (thymine ← → adenine) at position 343 of the alignment; GenBank accession numbers JX260254– JX260274). On average, the sequences showed base-pair frequencies of T: 38.0%, C: 18.5%, A: 26.3%, G: 17.2% (AT rich). 16S sequences were obtained from 35 macrostylid specimens resulting in a 385 bp alignment, with no single variable site (GenBank accession numbers JX260314– JX260348). Here, the sequences showed average base-pair frequencies of T: 31.5%, C: 16.4%, A: 35.3%, G: 16.9% (AT rich). The 12S dataset comprises the largest dataset. Sequences were obtained from 39 individuals resulting in a 503 bp alignment, with two closely related haplotypes (separated by two point mutations: transversion (adenine← → thymine) at position 88 of the alignment; transition (cytosine ← → thymine) at position 244; GenBank accession numbers JX260275– JX260313). For 12S, the sequences showed average base-pair frequencies of T: 33.9%, C: 18.0%, A: 31.4%, G: 16.7% (AT rich).

## Discussion

### Morphological Affinities

Eight species of Macrostylidae have previously been described from the Southern Ocean (Figure 1). *Macrostylis roaldi* sp. nov. shares the general appearance with *M. vinogradovae* Mezhov, 1992 and *M. setulosa* Mezhov, 1992 [68] with regard to the habitus, posterolateral margins and setation. The most obvious characters unique to *M. roaldi*, however, can be found in the prominent first sternal spine in both sexes as well as the rather short pleotelson and opercular pleopods in relation to body size. Moreover, the setation of all pereopods shows considerable differences. A sexual dimorphism affecting the posterolateral setae is found in *M. roaldi* that has never been reported before. However, only for a small number of species both sexes are known [73]. Background knowledge about sexual dimorphism in Macrostylidae is thus still scarce.

### Developmental and Reproductive Notes

For Haploniscidae, Wolff and Brökeland described the developmental trajectories of several species in detail [64], [76]. They found three manca stages and three male and female stages each. In Munnopsidae and various other janiroidean families, three manca stages in which the sex is not determinable, and slightly varying numbers of female (8) and male (2–3) stages have been repeatedly reported from [64], [76], [77]. It is a rare occasion to find all stages of a deep-sea isopod species and for Macrostylidae, not a single case has been reported. Despite the great sampling effort taken during BIOPEARL 2, not all stages were collected and it is thus not possible to explore the full developmental trajectory or demography of *M. roaldi* in detail. Environmental conditions (such as depth-related factors) differ between stations and this may cause developmental differences [77]. Size ranges amongst other characters are thus largely overlapping amongst the pooled individuals and the starting stage of the development of the males may differ.

Nevertheless, among the males, four distinct stages could be identified. For the females, however, the large size range of the second identified stage suggests that several stages have been overlooked and pooled. Developing oostegites in macrostylids are not expressed as external buds and Macrostylidae differ in this regard from their close relatives Desmosomatidae and Munnopsidae. This makes identification of preparatory females difficult. Detailed anatomical studies and dissections of the ovaries are needed but this is beyond the scope of this article.

Setal counts on pereopods have been regarded as allometric, i.e. increasing with body growth [39] and this pattern was found in *M. roaldi* as well. In *M. roaldi* however, we compared the setation of the pereopod III ischium dorsal lobes on the left and right sides within individuals and found 36% (17 specimens) to be asymmetrical with this regard. This is interesting especially because this region is often used for species identification. We hence suggest that for species identification more information should be applied than setal counts. In a juvenile (Fig. 12) male, we found the prominent seta on the ischial apex of the left pereopod absent. We assume this may be caused by a developmental error or an injury caused in an earlier stage. Analysis of more specimens is needed to solidify our speculation and elucidate the developmental trajectory of this species.

Dissection of one ovigerous female did not reveal developing oocytes in the gonads which suggests semelparity in *M. roaldi*. However, the small number of ovigerous specimens at hand does not allow adequate studies or final conclusions. The size range observed here for ovigerous females (3.2–3.8 mm) would allow multiple reproductive cycles. Any size difference could also be explained by potential effects of variation in the environment as the specimens originate from different stations.

### Distribution

The geographic and depth ranges recorded for *M. roaldi* (Fig. 1; Table 2) are remarkable given that a brooding mode of reproduction [78] and an infaunal lifestyle [41], [42], [45] should limit their dispersal capabilities. It is even more surprising as macrostylids have a very limited number of offspring (Riehl, personal observation; 8–10 eggs or embryos in marsupium of the two ovigerous *M. roaldi* specimens at hand (Fig. 5).

**Table 2:** Coordinates and sampling information for the type locality and further records of *Macrostylis roaldi* sp. nov.

Station name	Start trawl [decimal degrees]		Start trawl depth [m]	End trawl [decimal degrees]		End trawl depth [m]	Sampling date [d/m/y]
	latitude	longitude		latitude	longitude		
**Type locality**							
BIO03-EBS-1B	−71.79152	−106.21394	577.67	−71.78885	−106.21531	577.67	04/03/2008
BIO04-EBS-1A	−74.35975	−104.74595	1414.29	−74.36108	−104.73653	1413.5	06/03/2008
BIO04-EBS-1B	−74.35721	−104.752	1415.86	−74.358	−104.74252	1415.58	06/03/2008
BIO04-EBS-3A	−74.39845	−104.63215	504.29	−74.40009	−104.62462	489.65	07/03/2008
BIO04-EBS-3B	−74.40232	−104.61505	495.97	−74.40409	−104.6077	508.53	07/03/2008
**Further records**							
BIO05-EBS-1A	−74.11822	−105.83776	1478.92	−74.11962	−105.82882	1486.13	09/03/2008
BIO05-EBS-2A	−73.88016	−106.31654	1045.85	−73.88211	−106.30944	1113.97	09/03/2008
BIO05-EBS-3B	−73.97693	−107.41019	551.7	−73.97922	−107.40435	545.76	10/03/2008
BIO06-EBS-3A	−71.34713	−110.01329	481.11	−71.34438	−110.01328	478.14	12/03/2008

Previous studies on Southern-Ocean deep-sea isopods have shown that most species have been found at only one or a few locations; the species are regarded to be rare and endemic [32] or distributed in patches which, combined with little sampling effort at greater depth, created the illusion of rarity [79], [80]. Given the regular findings of *M. roaldi* across space, a common and relatively wide or a less patchy occurrence can be assumed, probably quite different from other species of the family in deeper water or when compared to Desmosomatidae and Nannoniscidae from the same area [53] (but see [81]). Sampling strategies revealing the actual distribution however, are currently lacking for *M. roaldi* as well as for most deep-sea species [80].

The realization of wide and disjunct occurrences of other benthic direct-developing invertebrates in the Southern Ocean (e.g. [82], [83]) has been attributed to a rafting mode of long-distance dispersal. Some even outranged the distribution of *M. roaldi* by far, e.g. a doridid sea-slug species (similar 16S haplotype separated by ∼6,200 km) [49] and a serolid isopod species (closely related COI haplotypes and microsatellites ∼2,000 km apart) [50]. Such dispersal events are probably rare but explainable on the background of certain attributes of lifestyle of the respective species. Usually, rafting on preferred food items or on structures used for egg-clutch deposition that are vulnerable to drifting is assumed for explanation [49], [50], [84].

Based on its morphology, we assume that *M. roaldi*, like probably all Macrostylidae, can be regarded a soft-sediment dweller that is unlikely to climb or hold on to potential rafting structures like algae or sponges. Instead, it digs in the top layer of the sediment. Such behavior was observed only for *M. spinifera* by Hessler and Strömberg [42]. Nevertheless, it is likely to be similar to other known species of the family on the basis of strong similarities in morphological features attributed to a burrowing or tubicolous lifestyle. Locomotory abilities are strongly correlated with morphology [42], [45]. This assumption is further supported by other morphological [40] as well as sampling evidence [85], [86]. We can hence regard rafting an implausible explanation for the wide distribution of *M. roaldi*. A drifting mode of dispersal, however, cannot generally be excluded. Brökeland [76] as well as Brix and co-workers [51] have shown that some janiroidean isopods must be capable to maintain connectivity between populations across long distances and physical (topographic) barriers. They found evidence for gene flow connecting two populations of a strictly non-natatory isopod from the South Atlantic abyss across a strong topographic barrier, the Walvis Ridge. Deep-sea currents have been suggested to facilitate migration and dispersal in abyssal benthic organisms [11], [52], [53], possibly even more benthic storms [87]. Instead of individual movement, bottom currents and other erosion-deposition events on the shelf may be much more an important factor to realize dispersal beyond individual locomotory range by passive translocation with soft sediments [87]. No morphological features have been identified in *M. roaldi* that could be related to active swimming. However, the cuticle of *M. roaldi* is translucent and therefore not heavily calcified. This characteristic might facilitate passive transport in bottom-water currents. Enhanced sampling effort and standardized application of integrative taxonomy (combining several sources of evidence, e.g. morphology and DNA) would help to clarify this picture.

### Genetic Structure

Across many benthic taxa in Antarctica, species have a wide distribution. Re-examinations by molecular means however, have often revealed a more complex picture. Species have been found to comprise several previously unrecognized lineages, ‘cryptic’ species or species complexes [8]–[12], [88], [89] (but see [90]). With two point mutations in the 12S and COI fragments and no variation at all in the 16S sequences across all *M. roaldi* samples, in our study molecular results are in accordance with morphological findings. The potential existence of cryptic species within the samples could be ruled out. The depth-differentiation hypothesis and the isolation-by-distance hypothesis could both be rejected. The homogenized gene pool across at least 1,000 m depth is an indicator for gene flow between shelf and slope. Beyond that, the lacking (mitochondrial) genetic diversity of *M. roaldi* in this area of the world cannot be explained by maintained gene flow alone. The assumption of a bottleneck scenario [91]–[93], probably accompanied with slow mutation rates, and a relatively recent colonization is necessary to explain the observed pattern. The absence of nucleotide variation might thus still show the consequences of recolonization following the Last Glacial Maximum around 14,500 years ago [57]. However, selective sweep [94] cannot be ruled out as an alternative explanation. This phenomenon is driven by maternally-transferred endosymbionts [95] causing selection to favor one mitochondrial variant over another.

### Evidence for Shelf Refuges?

The idea that Antarctic benthic fauna partially survived the last glacial period in refuges is now generally accepted. However, their locations are still a matter of debate and the same is true for potential mechanisms of the fauna to survive [5], [19], [35], [49], [96]–[98]. The data presented here allow inference of the presence of only one well-linked or recently spread population of *M. roaldi* in the sampled area, i.e. across several hundreds of kilometers from the inner to the outer shelf. Given the glaciological history of Pine Island Bay [75] and current strong environmental changes that influence the study area [34], [53], *M. roaldi* might represent either a pioneer species which emerged from greater depth or an in-situ survivor from past major glaciations.

Refuges have been mostly suggested to be located either at deeper bathyal or abyssal depth [34]. Yet, depth-related physiological barriers [22], [99], [100] may hinder migration across depth, especially for benthic organisms. The Antarctic, however, is known for a high degree of eurybathic taxa [101], which can be interpreted as adaptation to oscillation of glacial extensions [5]. As our data show that *M. roaldi* occurs across at least 1,000 m depth range, migratory capabilities of macrostylids amongst other deep-sea isopods (see e.g. [51], [76]) could be underestimated. Additionally, the polar-emergence hypothesis is in concordance with a bottleneck scenario regarding a founder effect. The fact that sampling at the shelf break and in deep bathyal depths did not yield any individuals belonging to this species does not exclude their possible existence there. Thus, *M. roaldi* might well have colonized the shelf from the abyss following the Last Glacial Maximum. However, as no abyssal material is available for this species from off Pine Island Bay and *M. roaldi* has never been reported from elsewhere, there is no evidence to either support or decline this theory.

Contrastingly, slope refuges are regarded as implausible due to frequent sedimentary cascades caused by protruding glaciers. Such is theorized to have wiped out most of the fauna [34], [35]. This was not necessarily true all around the continent as West and East Antarctic Ice Sheets showed great differences in their maximum extent as well as diachronous expansions and retreats [102] (and see [98]). There is undoubtedly strong evidence for glaciers having widely bulldozed sediment to the shelf break at Pine Island Bay [75], [103] making survival for the benthos down the slope difficult. Nevertheless, mass-wasting impact was mainly localized in canyons or gullies created by and concentrating down-slope cascades of melt water, sediment and rock during maximum extent of the glaciers. Such gullies have been found at the Pine Island Bay slope [75] and are characterized by valleys of 100–250 m depth with adjacent flanks and plateaus. Consequently during the Last Glacial Maximum, the slope was strongly structured featuring some areas of high and others of much lower impact, in the latter of which survival might have been easily possible (see [104]). Furthermore, Antarctic benthic fauna shows high resilience to periodic disturbance [98] and the possibility for shelf fauna to survive major glaciations on the slope can hence not be excluded. Sediment cascades down slope would promote bottlenecks through habitat fragmentation and partial habitat destruction. Given further the close proximity of the slope to the shelf plus the observed depth distribution of *M. roaldi*, the slope-refuge scenario may seem somewhat more likely than colonization from the abyss.

Alternatively, refuges may have existed in shelf pockets free from ice sheets or under the glaciers. The existence of ice-free refuges on the shelf has been repeatedly suggested [34], [35], [38], [96], [98] but biological data supporting this theory are scarce. Marine fauna has been found under glaciers up to hundreds of kilometers from the open sea [105]–[108] so survival is possible there under certain conditions. Glaciers decoupled from the sediment are a prerequisite for this theory. Furthermore, a marine environment, i.e. supply with saline and oxygenated sea water, is a required feature of a subglacial refuge. The same holds true, but probably to a smaller extent, for the advection of food items from open water [107] as macrostylids have been found to mainly rely on phytodetritus [109]. Parallels between the environmental conditions in such subglacial shelf refuges with those found in the deep sea or in marine caves [110], [111] are obvious, especially with regard to limited food availability and stable abiotic conditions [112]. So we even argue that in the practical absence of food influx, survival in shelf refuges under the ice would have been possible for especially undemanding and persistent small-sized organisms originating from deep-sea fauna, such as macrostylids.

Nevertheless, either as shelf pockets or subglacial refugia, life on the shelf during the Last Glacial Maximum would have been affected by extreme conditions and great reduction of available habitats. Populations were most likely fragmented and habitat size might have been reduced strongly [18]. In consequence, the mitochondrial genotypes could have reached fixation. Subsequent postglacial (re-) colonization of the surrounding shelf area would have happened since 14,500–10,000 years [57], [75]. That might not be sufficient to re-establish (mitochondrial) genetic diversity via chance mutations or secondary colonization from elsewhere (if a second population of this species survived). This scenario would provide an alternative explanation for the observed genetic structure in *M. roaldi*. Yet, it does not provide hints about where on the Amundsen Sea shelf such refuges could have existed.

Geophysical data suggest that the troughs on the inner shelf at Pine Island Bay, though possibly free from grounded ice sheets, were uninhabitable. They were under strong influence from subglacial melt water, sedimentation, gravel deposition and sliding ice [75], [113]. Regular sediment-laden plumes [75] would have had catastrophic effects on marine fauna there. Consequently, *M. roaldi* has most likely colonized these troughs following the glacial retreat rather than using them as a refuge. However, more data from adjacent subtidal, shelf, shelf-break and deep-sea areas are required to identify the full range of *M. roaldi*, its source population, potential sister species and thus possible refuges.

### Conclusions

*Macrostylis roaldi* sp. nov. occurs widely in Pine Island Bay, in a geographic as well as bathymetric sense. Across its currently known distribution, this species is lacking (mitochondrial) genetic variability. This could be attributed to a bottleneck, probably caused by their emergence from bathyal or abyssal depth (founder effect) or by a catastrophic climate event such as the last glacial period that brought the ancestor population to close extinction. In the absence of nucleotide variability, we further see evidence for a colonization of the Pine Island Bay shelf by this species that must have happened relatively recently, following the Last Glacial Maximum (i.e. since 14,500–10,000 years). The lack of genetic structure and missing knowledge about closely-related species do not allow inference of a potential refuge. Assessment of the current knowledge about the glaciological history of the area plus the available evidence for life under ice sheets led to the conclusion that all three potential survival scenarios, i.e. on the shelf or polar emergence from the bathyal or abyssal provide equally plausible explanations for the observed pattern.

## Materials and Methods

### Study Area

The study area (Pine Island Bay, eastern Amundsen Sea, Fig. 1) is approximately 450 km wide, reaching from the tip of the Pine Island Glacier to the shelf break. The inner shelf at Pine Island Bay is extremely rugged and characterized by deep channels and furrows shaped by previous glaciations and deglacations; the topography smoothens towards the outer shelf. It is further characterized by an average depth of 500 m, with some deep inner shelf troughs at about 1700 m depth. There is some geophysical evidence that during past glacial maxima ice sheets expanded to the shelf break and grounded there [75], [114]. The Amundsen shelf is periodically flooded by relatively warm Circumpolar Deep Water [56] that is one main reason for the dramatic ice loss of the Pine Island Glacier [115]. The topography, physical conditions and hydrography of this area have been discussed in detail elsewhere [56], [75], [116]. The continental slope, or bathyal, we define here as the benthic environment between the shelf break and the continental rise. The depths along the continental shelf break of the Amundsen Sea is on average 500 m, but varies from 400 to >600 m [116]. At the continental rise around 3,000 m depth, the slope levels off down to the abyss.

### Sampling and Fixation

This study is based on benthic samples collected during the BIOPEARL 2 (BIOdiversity, Phylogeny, Evolution and Adaptive Radiation of Life in Antarctica) project of the British Antarctic Survey with R/V *James Clark Ross* (JR 179) to the Amundsen Sea in 2008. In total, 36 samples were taken on the inner and outer shelf of Pine Island Bay, at the continental shelf break, slope and in abyssal depth. An epibenthic sledge sensu Brenke [117] was applied between 480 and 3,500 m depth. From eight of these stations (Fig. 1), *Macrostylis roaldi* sp. nov. could be reported. Samples were fixed in cooled (−20°C) 96% ethanol and preserved in the same medium.

### Taxonomy

Specimens were transferred to a glycerine-96% ethanol solution (1∶1) and subsequently to pure glycerine in order to prepare habitus illustrations and for dissections. *Methylene blue* and *Chlorazol black* were used for staining: from a highly concentrated solution of the respective stain in 96% ethanol, a small droplet was added to the specimen embedded in glycerine. The viscosity of the glycerine allows control over the staining process to avoid over staining. Once the preferred stain intensity was reached, the specimens were transferred to pure glycerine. Temporary slides after Wilson [118] were used for habitus illustrations. Line drawings were made using a *Leica DM2500* compound microscope with camera lucida and contrast interference and calibrated using a stage micrometer. To trace line drawings, vector graphics software (*Adobe Illustrator*, ver. CS4-5) was applied following the methods described by Coleman [119], [120]. All plates were prepared using *Adobe Photoshop* (ver. CS4).

Measurements are presented as ratios (to normalize differences in body size) and were prepared from line drawings following Hessler [121] and Riehl *et al.* [73] using the distance-measurement tool in *Adobe Acrobat Professional*. Ranges are provided where several specimens were measured. Terminology, measures, description with DELTA [122], [123] follow Hessler [121], Wilson [124], Kavanagh and Wilson [125], Riehl & Brandt [39] and Riehl *et al.* [73]. Characters were coded in DELTA following Sereno [126] with some modifications for improved readability. The list of implicit characters was slightly modified from Riehl *et al*. [73] and can be obtained from the first author upon request.

Appendages embedded in glycerine were not directly transferred to *Euparal* because these do not mix, but permanent slides were prepared with *Euparal* using the following method: Dissected parts were first transferred from glycerine to 70% denatured ethanol then to 96% denatured ethanol and then to a mixture of *Euparal* and 96% denatured ethanol (approximately 1∶1). Depending on the size of the fragments, parts were kept in the respective media for up to 30 minutes to ensure sufficient penetration. Finally, parts could be transferred easily to *Euparal*.

A *Carl Zeiss Leo 1525* microscope was used for SEM. SEM stubs, whole specimens and slides were deposited at the Zoological Museum, University of Hamburg, Germany, accession numbers have a ZMH-K prefix. Type material analyzed for comparison is listed in Table 3.

**Table 3:** Material of previously described Antarctic and South Atlantic Macrostylidae studied for comparison with *Macrostylis roaldi* sp. nov.

Species	Museum accession no	Type status
*M. abyssalis* Brandt, 2004	ZMH K-40284, ZMH K-40285	Holo- and paratypes
*M. angolensis* Brandt, 2004	ZMH K-40280, ZMH K-40281	Holo- and paratypes
*M. antennamagna* Riehl & Brandt 2010	ZMH (K-42168), ZMH (K-42169), ZMH (K-42171),ZMH (K-42172)	Holo- and paratypes
*M. cerritus* Vey & Brix, 2009	ZMH K-41431, ZMH K-41432, ZMH K-41433, ZMH K-41434	Holo- and paratypes
*M. gerdesi* (Brandt, 2002)	ZMH 39915, ZMH 39916	Holo- and paratypes
*M. longipedis* Brandt, 2004	ZMH 40278	Holotype
*M. longispinis* Brandt, 2004	ZMH K-40286	Holotype
*M. meteorae* Brandt, 2004	ZMH K-40282, ZMH K-40283, ZMH K-40698	Holo- and paratypes
*M. obscurus* (Brandt, 1992)	BM(NH) 1990∶39:1	Holotype
*M. robusta* Brandt, 2004	ZMH K-40276, ZMH K-40277, ZMH K-40295, ZMH K-40296,ZMH K-40297	Holo- and paratypes
*M. sarsi* Brandt, 1992	BM(NH) 1990∶40:1	Holotype
*M. uniformis* Riehl & Brandt 2010	ZMH (K-42172), ZMH (K-42173), ZMH (K-42174)	Holo- and paratypes

BM(NH)  =  British Museum of Natural History, London, UK; ZMH  =  Zoological Museum, University of Hamburg, Germany.

The distribution map was produced using GIS software ArcView 10.0 (ESRI, USA).

All specimens were analyzed for developmental stage, body size, and setal counts on the pereopod III ischium dorsal lobe to test for allometric relationships in these characters. Statistical correlations were tested with JMP 9.0 (SAS Institute Inc., USA). Specimens with damaged left or right pereopod III were excluded from the analyses.

### Molecular Methods

Samples were kept in cold conditions whenever possible. For DNA extraction, 2–3 pereopods were removed from one side of the body. The phenol-chloroform extraction method was applied. Three mitochondrial markers, cytochrome-c-oxydase subunit 1 (COI) as well as the ribosomal RNA small and large subunits (12S, 16S) were chosen because 1) they find applicability in the DNA barcode of Life program, 2) they have been widely applied in deep-sea isopod research and hence allow certain comparability and, 3) they have been found to be appropriate markers to infer phylogenetic relationships of isopods from the population to the genus level.

All three markers were amplified in a 10 µL reaction volume containing 0.25 µL BSA, 0.5 µL dNTP [2.5 mM each], 1 µL Bioline 10xNH4 reaction buffer, 0.3 µL of each primer [10 µM], 0.5 µL Biolase MgCl2 [50 mM], 0.1 µL Biolase DNA Pol [5 u/µL], 2 µL of template DNA and nuclease-free H2O. The same primer pairs (Table 4) were used for PCR and cycle sequencing (CS) respectively in 16S and 12S. For amplification of COI, M13-tailed primers based on dgLCO1490/dgHCO2198 were used. Here, for cycle sequencing M13 primers [127] were used. PCR and CS primers are listed in Tab. 4. The PCR temperature profile consisted of an initial denaturation at 95°C (5 min), followed by 34–36 cycles of denaturation at 95°C (30 s), annealing at 48°C (30 s) and extension at 72°C (45 s) followed by a final extension at 72°C (5 min).

**Table 4:** 12S, 16S and COI primers.

Primer name	Sequence [5′–3′]	Reference
16S SF	GACCGTGCTAAGGTAGCATAATC	(L. M. Tsang, pers. comm.)
16S SR	CCGGTCTGAACTCAAATCGTG	[134]
H13842-12S	TGTGCCAGCASCTGCGGTTAKAC	[135], [136]
L13337-12S	YCTWTGYTACGACTTATCTC	[135], [136]
dgLCO1490 (COI)	GGTCAACAAATCATAAAGAYATYGG	[137]
dgHCO2198 (COI)	TAAACTTCAGGGTGACCAAARAAYCA	[137]

For CS, 30 cycles of 95°C (30 s), 48°C (30 s) and 60°C (4 min) were applied. 2 µL of PCR product was analyzed for purity and size conformity by electrophoresis in a 1.5% agarose gel with ethidium bromide. Remaining PCR product was purified applying ExoSap-IT (USB). A 5x dilution of the enzyme was used and 2 µL of that solution were added to 8 µL PCR product (or 4 µL were added to 18 µL PCR product). Samples were incubated for cleanup at 37°C (30 min) and the enzyme was deactivated at 80°C (20 min). Cycle sequencing was performed in 10 µL volume containing 1 µL purified PCR product, 0.5 µL BigDye Terminator, 1.75µL Big Dye Terminator reaction buffer, 0.5 µL primer and nuclease-free water. Cycle sequencing products were cleaned up with the Sephadex G-50 (Sigma S-5897) method, dried and stored at −20°C until sequencing.

Sequences were managed, processed and quality-checked with the software Geneious [128]. Sequence alignment was performed with MAFFT (v6.717b) [129] implemented in Geneious. The alignment of COI was additionally optimized manually using MEGA 4 [130] with consideration of the amino-acid translation to check for pseudogenes [131], [132]. Alignments were checked for mutations by eye. Because of the absence of nucleotide variation among the specimens analyzed, no further analyses were conducted.

### Digital Archiving

This article is deposited at PubMedCentral and LOCKSS.

Molecular sequences are deposited in GenBank and BoLD [133] and access numbers are provided in Table 1.

### Nomenclatural Acts

The electronic version of this document does not represent a published work according to the International Code of Zoological Nomenclature (ICZN), and hence the nomenclatural acts contained in the electronic version are not available under that Code from the electronic edition. Therefore, a separate edition of this document was produced by a method that assures numerous identical and durable copies, and those copies were simultaneously obtainable (from the publication date noted on the first page of this article) for the purpose of providing a public and permanent scientific record, in accordance with Article 8.1 of the Code. The separate print only edition is available on request from PLoS by sending a request to PLoS ONE, Public Library of Science, 1160 Battery Street, Suite 100, San Francisco, CA 94111, USA along with a check for $10 (to cover printing and postage) payable to “Public Library of Science”.

In addition, this published work and the nomenclatural acts it contains have been registered in ZooBank, the proposed online registration system for the ICZN. The ZooBank LSIDs (Life Science Identifiers) can be resolved and the associated information viewed through any standard web browser by appending the LSID to the prefix “http://zoobank.org/”. The LSID for this publication is: urn:lsid:zoobank.org:pub:1113243A-0A9F-4FBF-8739-BF255C4C8C8B.
